# Ostéosarcome primitif de la cuisse: à propos d'un cas

**DOI:** 10.11604/pamj.2015.20.210.5740

**Published:** 2015-03-06

**Authors:** Boubacar Zan Traoré, Anis Benmansour, Omar Saoud, Safia El Aboudi, Abdesslam Bouktab

**Affiliations:** 1 Clinique Chirurgicale Viscérale "K", Institut Nationale d'Oncologie, CHU Ibn Sina, Rabat, Maroc

**Keywords:** Cuisse, extra squelettique, ostéosarcome, primitif, thigh, Extra skeletal, osteosarcoma, primitive

## Abstract

À travers cette observation d'ostéosarcome extra-osseux de localisation atypique, le diagnostic clinique et radiologique, ainsi que le profil épidémiologique de cette entité pathologique sont discutés. Patient de 64 ans consultait pour tuméfaction évolutive douloureuse de la cuisse droite qui évoluait depuis plus de 03 ans. Ce patient avait bénéficié d'une TDM, d'une IRM puis d'une scintigraphie osseuse. Une exérèse chirurgicale de la tumeur avait été réalisée. L'analyse histopathologique de la pièce opératoire concluait à un ostéosarcome conventionnel extra osseux. Le patient a été adressé en oncologie pour complément de prise en charge. L'ostéosarcome extra squelettique occupe typiquement une situation profonde, sans lien avec l'os ou le périoste adjacent. Le pronostic est mauvais.

## Introduction

L'ostéosarcome extra squelettique est une néoplasie maligne rare (1% des sarcomes des tissus mous, 2 à 5% des ostéosarcomes) [1] et habituellement très agressive, touchant les individus au-delà de cinquante ans et prédominant au niveau des tissus mous des membres ou du rétro péritoine. Elle forme une substance ostéoïde et parfois du cartilage [1]. À travers cette observation d'ostéosarcome extra-osseux de localisation atypique, le diagnostic clinique et radiologique, ainsi que le profil épidémiologique de cette entité pathologique sont discutés.

## Patient et observation

**Figure 1:**
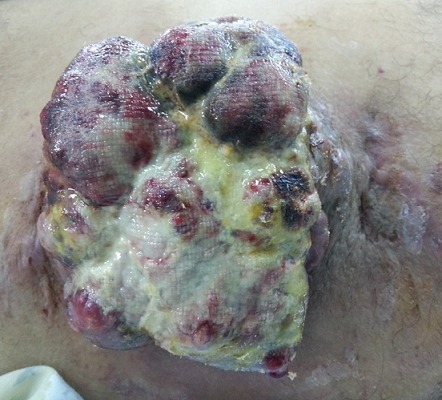
Aperçu de la masse à l'inspection Clinique

**Figure 2:**
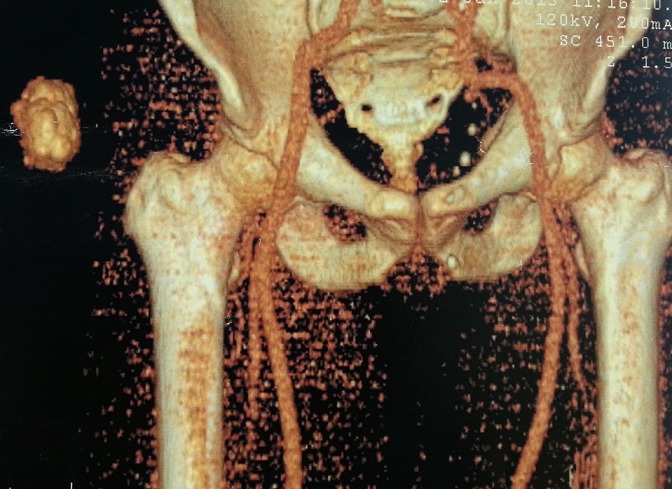
Coupe scannographique avec reconstruction VRT montrant un processus tissulaire des parties molles superficielles en regard de la hanche droite

**Figure 3:**
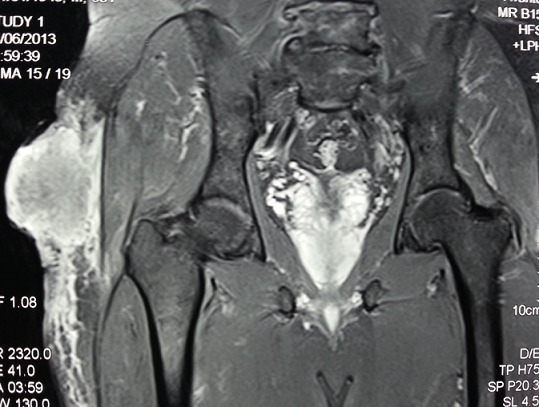
Coupe coronale après injection de gadolinium, un processus tumoral malin des parties molles superficielles de la hanche droite hyposignal T1 hypersignal T2 et fortement après Gado et lisère de séparation avec le muscle grand fussier

**Figure 4:**
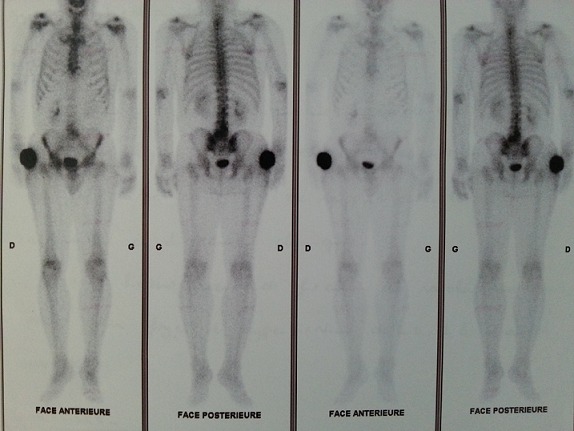
Scintigraphie osseuse du corps entier mettant en évidence une hyperfixation intense extra osseuse en regard de la cuisse droite en rapport avec la masse retrouvé à l'examen clinique

Patient de 64 ans, sans antécédent pathologique notable, consultait pour une tuméfaction douloureuse de la cuisse droite qui évoluait depuis plus de 03 ans et qui augmentait progressivement de taille. L'examen physique avait retrouvé une masse bourgeonnante, mobile, présentant des remaniements nécrotiques et hémorragiques, douloureuse, mesurant 14×12,5×4 cm (Figure 1). Les radiographies du fémur et de la cuisse montraient une masse des tissus mous, avec calcification en regard du grand trochanter droit. Une TDM thoraco- abdomino-pelvienne et de la cuisse droite avait objectivé un épaississement de la peau et du tissu sous cutané péritrochantérien et fessier du côté droit, associé à quelques calcifications para trochantériennes sans image nodulaire suspecte (Figure 2). Absence de lésion osseuse visible. Une IRM était réalisée en coupes axiales et coronales T1, T2, et DP, SE et Fat-Sat et T1 Fat-Sat après injection de Gado permettait d'objectiver un processus tumoral d'allure maligne des parties molles superficielles de la hanche droite, types liposarcome (Figure 3). Une scintigraphie osseuse à l'HMDP-99mTc réalisée avait mis en évidence une hyperfixation intense extra osseuse en regard de la cuisse droite en rapport avec la masse retrouvée à l'examen clinique. Aucune autre lésion ostéoblastique loco-régionale ou à distance n'avait été objectivée (Figure 4). Le patient avait bénéficié d'une exérèse tumorale chirurgicale large prenant en profondeur une pastille musculaire au dépend des muscles grand fessier et tenseur du fascia lata, à 2cm du plan profond musculaire, à 2,5cm de la limite supérieure, à 4cm de la limite postérieure, à 1,8cm de la limite inférieure et à 7cm de la limite antérieure. L'analyse histopathologique de la pièce opératoire concluait à un aspect morphologique d'un ostéosarcome conventionnel extra osseux de 12,5cm de grand axe (absence de lésion squelettique sur la radiologie); absence d'embole vasculaire néoplasique; plan profond indemne passe à 1,5cm; limites latérales saines, la plus proche (la limite inférieure) passe à 1,8cm. Le patient a été adressé en oncologie, après cicatrisation, pour une poly-chimiothérapie adjuvante. Neuf mois après l'exérèse chirurgicale de la tumeur, il n'existait pas de récidive locale ni de métastase décelable.

## Discussion

L'ostéosarcome extra squelettique ou extra osseux est une tumeur des tissus mous, qui est par définition libre du squelette et composée de cellules malignes ostéoblastiques produisant de la matrice osseuse [2]. Cette néoplasie rare représente environ 1% des sarcomes des tissus mous et moins de 5% des ostéosarcomes. Contrairement à l'ostéosarcome squelettique, présent dans les deux premières décennies de vie, l'ostéosarcome extra-squelettique affecte les individus de plus de 50 ans. Cette tumeur est plus fréquemment rencontrée chez l'homme que chez la femme avec un ratio de 1,9.-1 [3].

La plupart des ostéosarcomes extra-squelettiques ont fermement attachés au facia musculaire. Les localisations les plus fréquentes sont les tissus mous des membres et le rétro-péritoine [4, 5]. Toutefois, la tumeur peut survenir dans n'importe quelle partie du corps, y compris le cœur, le cerveau, le poumon, le médiastin, le sein, le foie, le colon, l’œsophage, le diaphragme, la langue, les mains, la peau, le cuir chevelu, la mandibule et le pénis. Plusieurs facteurs de risque ont été rapportés dans la littérature, notamment les radiations ionisantes et les traumatismes. Des ostéosarcomes extra-squelettiques survenant sur une lésion de myosite ossifiante ou sur un foyer d'ossification hétérotopique ont été décrits. L'ostéosarcome squelettique évolue de manière agressive avec 70% de récidive locale et 80% de métastases deux ans après exérèse chirurgicale de la tumeur primaire. La survie médiane est de 24 mois avec un taux de survie à 5 ans de 13 à 37% [6].

L'exérèse chirurgicale complète offre le meilleur espoir de guérison. La chimiothérapie adjuvante s'est avérée curative chez certains patients après exérèse complète sans résidu néoplasique macroscopique, et offre une palliation chez les patients présentant une maladie métastatique ou inopérable. Comme l'ostéosarcome classique, l'ostéosarcome squelettique présente d'importantes variations histopathologiques et peut par endroit ressembler à un fibrosarcome, à un Schwannome malin ou à histiocytome fibreux. Il en découle que l'analyse histopathologique des pièces de ponction-biopsie peut se révéler trompeuse, alors que l'exérèse chirurgicale à des fins diagnostiques de la lésion entière est souvent impossible.

## Conclusion

L'ostéosarcome extra-squelettique occupe typiquement une situation profonde, sans lien avec l'os ou le périoste adjacent. Le pronostic est mauvais. Plus de 80 à 90% des patients développent une récidive locale et des métastases, notamment pulmonaire. Étant une tumeur exceptionnelle, l'ostéosarcome primitif de la cuisse doit être pris en charge en réunion de concertation multidisciplinaire par une équipe spécialisée.
